# Melioidosis, Singapore, 2003–2014

**DOI:** 10.3201/eid2401.161449

**Published:** 2018-01

**Authors:** Long Pang, Patrick N.A. Harris, Rachel L. Seiler, Peng Lim Ooi, Jeffrey Cutter, Kee Tai Goh, Alex R. Cook, Dale Fisher, Louis Yi Ann Chai

**Affiliations:** National University of Singapore, Singapore (L. Pang, P.N.A. Harris, P.L. Ooi, J. Cutter, K.T. Goh, A.R. Cook, D. Fisher, L.Y.A. Chai);; National University Health System, Singapore (L. Pang, P.N.A. Harris, P.L. Ooi, J. Cutter, K.T. Goh, A.R. Cook, D. Fisher, L.Y.A. Chai);; University of Queensland, Brisbane, Queensland, Australia (P.N.A. Harris, R.L. Seiler);; Ministry of Health, Singapore (P.L. Ooi, J. Cutter, K.T. Goh)

**Keywords:** *Burkholderia pseudomallei*, diabetes, pneumonia, abscess, mortality, melioidosis, Singapore, bacteria, Australia, Thailand

## Abstract

In contrast with northern Australia and Thailand, in Singapore the incidence of melioidosis and co-incidence of melioidosis and pneumonia have declined. *Burkholderia pseudomallei* deep abscesses increased 20.4% during 2003–2014. These trends could not be explained by the environmental and climatic factors conventionally ascribed to melioidosis.

Melioidosis is endemic throughout Southeast Asia and northern Australia. Caused by the environmental gram-negative bacillus *Burkholderia pseudomallei*, melioidosis can manifest as bacteremia, abscesses, or pneumonia. A case-fatality rate of >50% has been reported (*1*).

Australia, Thailand, and Singapore have the most reported cases of melioidosis worldwide (*2*). The geographic, climatic, and infrastructural landscapes of these 3 countries differ vastly. The population demographics and ease of accessibility to healthcare also differ and may influence disease manifestations and outcomes. Epidemiologic case studies from Singapore are limited (*3*). We describe the latest epidemiologic findings based on national melioidosis surveillance data for 2003–2014.

## The Study

Melioidosis is a notifiable condition in Singapore. The laboratory and clinical criteria for notification are defined by the Ministry of Health, Singapore (*4*). Our study comprised all cases reported by medical practitioners and microbiology laboratories during 2003–2014. Public health officers collected demographic and clinical details of persons with notified cases using a standardized template. The data were extracted from the Ministry’s Communicable Disease Surveillance system (*5*). We used a Poisson regression model, offset for population, to fit the annual melioidosis incidence to measure overall trend. A binomial generalized linear model with identity link was used to quantify temporal changes in the absolute proportion of melioidosis cases having each co-morbidity. We conducted a binomial test of whether the prevalence of comorbidities equaled that of the general population; the latter was derived from the last published National Health Survey 2010 (http://www.moh.gov.sg/content/moh_web/home/Publications/Reports/2011/national_ health_survey2010.html) for persons 50−59 years of age (for diabetes and hypertension) and 40−54 years of age (for renal impairment). Analyses were performed with R Statistical Software version 3.3.1 (https://www.r-project.org/).

During the 12 years studied, notifications were received for 614 (range 31–96 annually) melioidosis cases (Table). The mean age of patients was 51.4 years; most (84.0%) patients were males. Most (72.1%) cases occurred in patients >45 years of age. The overall incidence of melioidosis was 1.1 per 100,000 population; incidence was highest among Malay and Indian populations (2.4 and 2.1/100,000, respectively). The most common manifestations of melioidosis were *B. pseudomallei* bacteremia (60.3%), abscesses (40.7%), and pneumonia (33.1%). Among the co-morbidities reported (Table), diabetes (56.7%) and renal impairment (15.3%) had significantly higher prevalence among persons with melioidosis than among the general population of approximately the same age (19.3%, p<0.001 for diabetes; 2.0%, p<0.001 for renal impairment). In contrast, hypertension prevalence was consistent between melioidosis patients and the general population (35.5% vs. 31.9%; p = 0.057).

**Table:** Demographic characteristics of patients with melioidosis, Singapore, 2003–2014*

Characteristic	2003	2004	2005	2006	2007	2008	2009	2010	2011	2012	2013	2014	Total (%)†
No. cases	42	96	74	59	57	60	37	58	34	31	34	32	614
Patient age, y													
Mean	52.6	51.3	51.2	50.6	56.9	49.6	54.1	55	46.1	45.9	53.7	50	
Minimum	19	7	0	14	9	3	13	10	8	12	9	13	
Maximum	82	91	95	85	87	77	83	87	77	76	88	83	
Sex													
F	6	15	9	13	7	9	8	13	6	1	10	1	98 (16.0)
M	36	81	65	46	50	51	29	45	28	30	24	31	516 (84.0)
Incidence by race‡													
Chinese	0.9	2.3	1.3	1.3	0.9	1.3	0.7	1	0.5	0.4	0.5	0.4	1.0§
Malay	2	2.9	4.1	2.2	3.5	2.8	0.8	3.2	1.6	1.8	2.3	1.2	2.4§
Indian	0.7	3.8	3.2	2.2	4.5	2.5	1.5	0.6	1.1	1.4	1.1	2.3	2.1§
Comorbidity													
Diabetes mellitus	19	61	43	31	33	39	19	38	20	15	18	12	348 (56.7)
Hypertensive disease	10	29	23	18	27	27	8	21	17	10	13	15	218 (35.5)
Hyperlipidemia	1	2	10	7	4	11	2	22	4	5	7	12	87 (14.2)
Ischemic heart disease	3	19	5	10	4	4	1	3	2	1	3	5	60 (9.8)
Chronic liver disease/cirrhosis	0	4	1	1	1	1	0	0	1	0	0	0	9 (1.5)
Neoplasm	0	0	5	34	0	0	0	5	0	1	0	0	45 (7.3)
Renal impairment	9	23	11	10	13	12	3	3	3	1	3	3	94 (15.3)
Anemia	1	3	4	1	2	0	0	0	0	0	1	0	12 (2.0)
Tuberculosis	0	10	5	0	2	0	0	2	0	1	1	0	21 (3.4)
COPD	0	2	1	1	1	1	0	0	0	0	0	0	6 (1.0)
Asthma	0	4	6	1	1	2	0	2	1	1	0	0	18 (2.9)
No. deaths, %	6 (14.3)	25 (26.0)	12 (16.2)	9 (15.3)	12 (21.1)	12 (20.0)	5 (13.5)	14 (24.1)	6 (17.6)	2 (6.5%	8 (23.5)	2 (6.3)	113 (18.4)

*Data extracted from (*5,9)*. COPD, chronic obstructive pulmonary disease. †Values are no. (%) unless otherwise indicated. ‡These are the standard reported races/ethnicities in Singapore. Incidence is per 100,000 population. §Mean.

**Figure 1:**
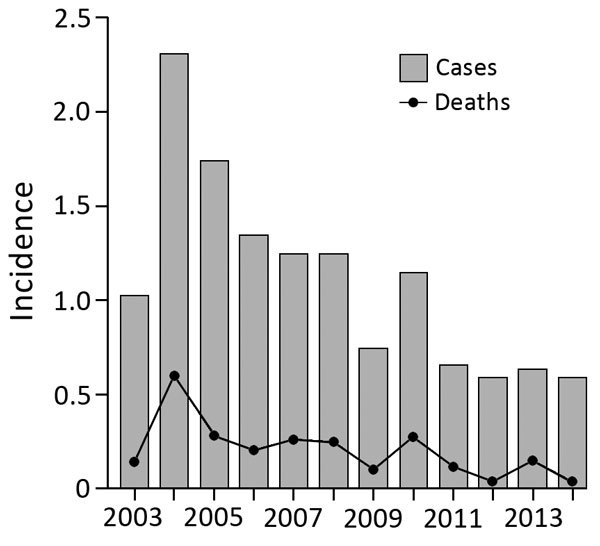
Annual incidence of melioidosis per 100,000 persons, Singapore, 2003–2014.

**Figure 2:**
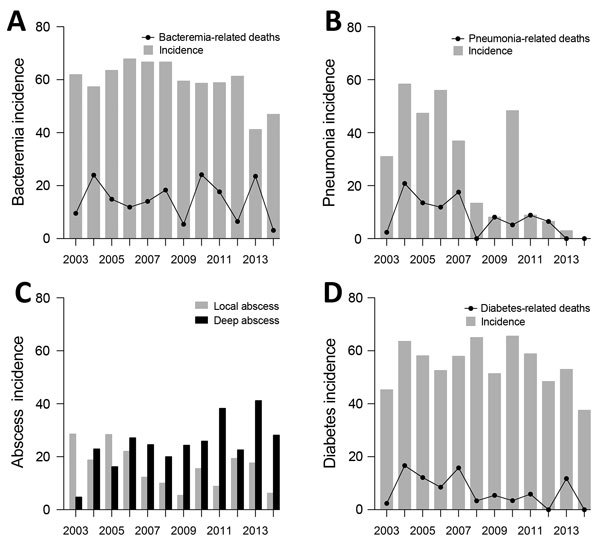
Melioidosis trends, Singapore, 2003–2014. A) Annual incidence of *Burkholderia pseudomallei* bacteremia and bacteremia-related deaths per 100 melioidosis cases. B) Annual incidence of melioidosis patients with pneumonia and pneumonia-related death per 100 melioidosis patients. C) Annual incidence of *B. pseudomallei* abscesses per 100 melioidosis patients. Local abscesses as per superficial or cutaneous, and deep abscess as per deep organ (primarily liver, spleen and prostate). D) Annual incidence of diabetes and diabetes-related death per 100 melioidosis patients.

During 2003–2014, melioidosis incidence decreased by 10% annually (incidence rate ratio 0.900; 95% CI 0.878–0.923; p<0.001). The overall death rate was 18.4% and decreased by 12.3% annually (incidence rate ratio, 0.877; 95% CI 0.826–0.931; p<0.001) (Figure 1). Among melioidosis patients, there was a borderline declining trend in *B. pseudomallei* bacteremia cases annually (absolute risk reduction [ARR] 1.1%; 95% CI −2.3% to 0.1%; p = 0.076). However, the death rate for bacteremic patients did not change (ARR 0.4%; 95% CI −1.2% to 0.4%; p = 0.355) (Figure 2, panel A). In contrast, melioidosis patients with pneumonia decreased from a peak of 56 cases in 2004 to no cases in 2014 (ARR 5.1%; 95% CI −5.6% to −4.6%; p<0.001), accompanied by an ARR of 1.5% in mortality annually (95% CI −1.9% to −1.1%; p<0.001) or 18% reduction in death during the 12-year period (Figure 2, panel B).

The annual incidence of *B. pseudomallei* abscesses was unchanged during 2003–2014, but the number of patients with deep abscesses (primarily liver, spleen, or prostate) increased (absolute risk increment 1.7% annually; 95% CI 0.7%–2.8%; p = 0.001) or 20.4% increment over 12 years (Figure 2, panel C). We saw no evidence of a change in the prevalence of diabetes among persons with melioidosis (ARR 0.7%; 95% CI −1.9% to 0.5%; p = 0.277), although there was an ARR of 0.9% in mortality annually (95% CI −1.5% to −0.4%; p = 0.002) or 10.8% reduction over 12 years, for this at-risk patient group (Figure 2, panel D).

This study, comprising one of the largest melioidosis patient cohorts thus far, yielded some unanticipated disease trends from Singapore. Foremost was the decreasing melioidosis incidence, in the context of declining *B. pseudomallei* bacteremia cases and, more significantly, the steady decrease in pneumonia.

By contrast in Australia and Thailand, melioidosis numbers stayed constant or increased (*6*). Pneumonia continued to feature prominently in 50% of the cases in these countries (*7*). It would be reasonable to theorize that pneumonia would be the predominant feature following inhalation of aerosolized *B. pseudomallei* after rainfall (*8*). Several groups have reported the association between rainfall, humidity, and water exposure with melioidosis (*9*,*10*). In equatorial Singapore, however, rainfall patterns have not changed recently. In addition, our analysis of rainfall and humidity during the corresponding study period found these climatic variables to be constant on a yearly scale (rainfall, p = 0.846; humidity, p = 0.815). Hence, climate is unlikely to be related to the decline in disease incidence.

Soil, in particular anthrosol and acrisol soil, encountered in irrigated agriculture, has been suggested as a likely reservoir for *B. pseudomallei* (*10*). Consequently, urbanization leading to reduction of agricultural or rural land areas ought to align with a lower incidence of melioidosis. However, the developed island state of Singapore already had reached 100% urbanization by the early 1990s (*11*); thus, urbanization could not have been the primary factor for this decreasing incidence after 2003.

Conversely, water sanitization, storm/rainwater drainage, and flood reduction have remained a major focus of ongoing infrastructural improvements of the Singapore government through the 2000s to now. The national water strategy entails a complex system encompassing optimized drainage and collection, followed by water treatment intended for consumption and use. Flood risk is managed through design and implementation of state-of-the-art water drainage systems and flood-protection measures for public infrastructures (*12*). Together with a national flood alert response plan, these measures potentially minimize direct rain or contaminated water exposure and aerosol inhalation risk; thus, they are plausible factors to account for the overall melioidosis and pneumonia case reductions, but further study is needed to investigate this possibility.

We did not anticipate the 20.4% increase of *B. pseudomallei* deep organ abscesses during the study period. We theorized that it might partly be explained by compromised host immunity to *B. pseudomallei*, attributable to the progressively higher prevalence of diabetes in Singapore (than in Australia and Thailand) (*13*). Specifically in the context of abscess development, diabetic tissue macrophages impair *B. pseudomallei* killing capacity and an impaired interleukin IL12–interferon-γ response had been implicated (*14*). Conversely, melioidosis-associated deaths in patients with diabetes had decreased by 10.8% during our study period. This improvement in outcome might have found its roots in the drive for optimization of diabetes care at the public health level in Singapore in recent years (*15*).

## Conclusions

The overall death rate from melioidosis in Singapore was 18.4%, similar to that in the Northern Territory of Australia (14%). In both locations, these rates were attained on the background of similar standards and accessibility to healthcare and a low threshold for institution of treatment for melioidosis in accordance with recommendations (*1*). As efforts continue to further optimize clinical outcomes in acute melioidosis, our experience from Singapore for 2003–2014 suggests that acquisition of melioidosis and pneumonia may be curtailed through enhanced environmental and water management incorporating countrywide infrastructural improvements. In addition, enhanced management of the at-risk cohort of persons with diabetes also might prove pivotal in reducing disease.

## References

[1] Wiersinga WJ, Currie BJ, Peacock SJ. Melioidosis. N Engl J Med. 2012;367:1035–44. 10.1056/NEJMra1204699 22970946

[2] Nasner-Posso KM, Cruz-Calderón S, Montúfar-Andrade FE, Dance DA, Rodriguez-Morales AJ. Human melioidosis reported by ProMED. Int J Infect Dis. 2015;35:103–6. 10.1016/j.ijid.2015.05.009 25975651 PMC4508390

[3] Heng BH, Goh KT, Yap EH, Loh H, Yeo M. Epidemiological surveillance of melioidosis in Singapore. Ann Acad Med Singapore. 1998;27:478–84. 9791650

[4] Ministry of Health Singapore. Melioidosis. In: Singapore Ministry of Health and Tan Tock Seng Hospital, editor. A guide on infectious diseases of public health importance in Singapore. Singapore: Ministry of Health Singapore; 2004. p. 55–6.

[5] Ministry of Health. Singapore. Communicable diseases surveillance in Singapore. 2015 [cited 2016 Jan 5]. https://www.moh.gov.sg/content/moh_web/home/Publications/Reports/2015/communicable-diseases-surveillance-in-singapore-2014.html

[6] Limmathurotsakul D, Wongratanacheewin S, Teerawattanasook N, Wongsuvan G, Chaisuksant S, Chetchotisakd P, et al. Increasing incidence of human melioidosis in Northeast Thailand. Am J Trop Med Hyg. 2010;82:1113–7. 10.4269/ajtmh.2010.10-0038 20519609 PMC2877420

[7] Meumann EM, Cheng AC, Ward L, Currie BJ. Clinical features and epidemiology of melioidosis pneumonia: results from a 21-year study and review of the literature. Clin Infect Dis. 2012;54:362–9. 10.1093/cid/cir808 22057702 PMC3258273

[8] Lim C, Peacock SJ, Limmathurotsakul D. Association between activities related to routes of infection and clinical manifestations of melioidosis. Clin Microbiol Infect. 2016;22:79.e1–3. 10.1016/j.cmi.2015.09.016 26417852 PMC4721533

[9] Liu X, Pang L, Sim SH, Goh KT, Ravikumar S, Win MS, et al. Association of melioidosis incidence with rainfall and humidity, Singapore, 2003-2012. Emerg Infect Dis. 2015;21:159–62. 10.3201/eid2101.140042 25531547 PMC4285244

[10] Limmathurotsakul D, Kanoksil M, Wuthiekanun V, Kitphati R, deStavola B, Day NP, et al. Activities of daily living associated with acquisition of melioidosis in northeast Thailand: a matched case-control study. PLoS Negl Trop Dis. 2013;7:e2072. 10.1371/journal.pntd.0002072 23437412 PMC3578767

[11] Institute of Southeast Asian Studies. Urbanisation in Southeast Asian Countries. 2010 [cited 2016 Aug 3]. https://www.iseas.edu.sg/images/centres/asc/pdf/UrbanSEAsia-prelimasof13Jul10.pdf

[12] Public Utilities Board, Singapore National Water Agency. Stormwater management. 2016 [cited 2016 Aug 3]. https://www.pub.gov.sg/drainage/stormwatermanagement

[13] Phan TP, Alkema L, Tai ES, Tan KH, Yang Q, Lim WY, et al. Forecasting the burden of type 2 diabetes in Singapore using a demographic epidemiological model of Singapore. BMJ Open Diabetes Res Care. 2014;2:e000012. 10.1136/bmjdrc-2013-000012 25452860 PMC4212579

[14] Tan KS, Lee KO, Low KC, Gamage AM, Liu Y, Tan GY, et al. Glutathione deficiency in type 2 diabetes impairs cytokine responses and control of intracellular bacteria. J Clin Invest. 2012;122:2289–300. 10.1172/JCI57817 22546856 PMC3366396

[15] Toh MP, Leong HS, Lim BK. Development of a diabetes registry to improve quality of care in the National Healthcare Group in Singapore. Ann Acad Med Singapore. 2009;38:546–6. 19565107

